# Influenza Vaccination Coverage Among Pregnant Women — United States, 2012–13 Influenza Season

**Published:** 2013-09-27

**Authors:** Sarah Ball, Sara Donahue, David Izrael, Deborah K. Walker, Rachel Martonik, Charles DiSogra, Helen Ding, Stacie M. Greby, Katherine Kahn, Peng-Jun Lu, Walter W. Williams, James A. Singleton, Erin D. Kennedy, Carolyn B. Bridges, LisaA. Grohskopf, Denise J. Jamieson, Indu Ahluwalia

**Affiliations:** Abt Associates, Inc., Cambridge, Massachusetts; Abt SRBI, New York, New York; Immunization Svcs Div; Influenza Div, National Center for Immunization and Respiratory Diseases; Div of Reproductive Health, National Center for Chronic Disease Prevention and Health Promotion, CDC

Pregnant women and infants aged <6 months are at increased risk for influenza-related severe illness and hospitalization. Influenza vaccination of pregnant women has been shown to reduce the risk for illness in both mother and infant (1). To help protect pregnant women, the Advisory Committee on Immunization Practices (ACIP) and the American College of Obstetricians and Gynecologists recommend influenza vaccination for all women who are or will be pregnant during the influenza season, regardless of trimester (1,2). To estimate influenza vaccination coverage among pregnant women during the 2012–13 influenza season, CDC analyzed data from an Internet panel survey conducted April 1–12, 2013. Among 1,702 self-selected survey respondents pregnant at any time during the 4-month period of October 2012–January 2013, 50.5% reported they received influenza vaccination before or during their pregnancy. Influenza vaccination coverage was higher among women reporting both a health-care provider recommendation and offer of influenza vaccination (70.5%) compared with women who received a recommendation but no offer of vaccination (46.3%) and women who received no recommendation (16.1%). Vaccination coverage of women who will be or are pregnant during an influenza season might be improved by implementing a combination of community-based interventions, including enhanced access to low-cost vaccination services, provider recommendation and offer of influenza vaccination, and education of pregnant women about influenza vaccination safety and efficacy during pregnancy to increase demand (3).

To provide end-of-season estimates of influenza vaccination coverage, health-care provider recommendation and offer of vaccination, and information on knowledge, attitudes, and behaviors related to influenza vaccination among women pregnant during the 2012–13 influenza season, before the 2013–14 influenza season, CDC conducted an Internet panel survey during April 1–12, 2013.* Women aged 18–49 years who were pregnant at any time since August 2012 were recruited from a SurveySpot panel, a general population opt-in Internet panel operated by Survey Sampling International.† Of 6,633 women who entered the survey, 2,198 were determined to be eligible, and 2,047 (93.1%) completed the survey.§ Data were weighted to reflect the age groups, race/ethnicity, and geographic distribution of the total U.S. population of pregnant women during 1990–2008.¶ The methods and questions used in the April 2013 survey were similar to the April 2011 and April 2012 surveys (4,5). However, for this analysis, vaccination status was defined differently from the analyses of the 2010–11 and 2011–12 influenza seasons: 1) the vaccination time frame changed to July through April, compared with the previous timeframe of August through April; and 2) a woman was considered vaccinated only if she was vaccinated before or during pregnancy, whereas previously women vaccinated after pregnancy had also been counted (4,5). In this analysis, the study population was limited to women reporting being pregnant any time during the usual peak influenza vaccination period of October–January (n = 1,702).

Survey respondents were asked questions about 1) their vaccination status before and during pregnancy, 2) whether their health-care provider recommended and offered influenza vaccination, 3) their attitudes regarding influenza and influenza vaccination, and 4) their reasons for receiving or not receiving influenza vaccination. To simplify the analysis, responses to five individual questions on attitudes were used to develop two composite scores defining attitudes toward influenza vaccination efficacy and the safety of influenza vaccination. A response to a sixth question was used as a measure of concern about influenza infection.** Because the study sample was based on pregnant women from an opt-in Internet panel rather than a probability sample, no statistical tests were performed. Differences were noted when there was a difference of ≥5 percentage points between any values being compared.

Of the 1,702 women pregnant at any time during October 2012–January 2013, 50.5% reported influenza vaccination since July 1, 2012; 14.6% were vaccinated before pregnancy and 35.9% during pregnancy (15.7% first trimester, 10.6% second trimester, 8.1% third trimester, and 1.5% unknown trimester) (Table 1). Among the 1,620 women with at least one health-care provider visit since July 2012 who provided information on a provider recommendation and offer, 54.6% reported receiving a provider recommendation and offer of vaccination, 16.7% reported receiving a provider recommendation but no offer of vaccination, and 28.7% reported receiving no recommendation. Women who reported receiving both a provider recommendation and offer of influenza vaccination had higher vaccination coverage (70.5%) compared with women who reported receiving a provider recommendation but no offer (46.3%) and women who reported receiving no recommendation (16.1%) (Table 1, Figure). Women with the following reported characteristics had lower influenza vaccination coverage than other women within each comparison stratum: aged 18–24 years, non-Hispanic black, having an education less than a college degree, not married, reporting no health insurance, not working for wages, living below the poverty level, having no high-risk conditions associated with increased complications for influenza, and having fewer than six health-care provider visits since July 2012 (Table 1). Vaccination coverage among women with a negative attitude toward the efficacy of influenza vaccination was 9.8%, compared with 64.2% among those with a positive attitude. Women with a negative attitude towards the safety of vaccination had lower coverage than those with a positive attitude (13.0% versus 65.6%), and those with no concern about influenza infection had lower coverage than those with concern about influenza infection (47.1% versus 52.8%) (Table 1). The outcomes regarding attitudes were similar whether using responses to the composite scores or the individual questions.

Overall, 72.3% of women reported receiving a health-care provider recommendation for vaccination, with or without reporting an offer of vaccination (Table 2). Women with both a provider recommendation and offer of influenza vaccination had higher vaccination coverage compared with women who received only a recommendation or who received no recommendation across all socio-demographic subgroups and attitude categories (Table 2). Among women who received a provider recommendation and offer of vaccination, coverage was 19.4% for those who reported a negative attitude toward influenza vaccination efficacy, 19.4% for those who reported a negative attitude towards the safety of influenza vaccination, and 68.8% for those who reported no concern about influenza infection; vaccination coverage was lower among women who did not receive a provider recommendation and also reported a negative attitude toward vaccination efficacy (2.5%) or the safety of influenza vaccination (7.7%) or no concern about influenza infection (15.6%).

The top three reasons women reported for vaccination were to protect their infant from influenza (33.2%), to protect themselves from influenza (20.0%), and because their health-care provider recommended vaccination (15.7%). The top three reasons reported for nonvaccination were concern about safety risk to the infant (20.5%), that the vaccination would give pregnant women influenza (13.6%), and that vaccination was not effective in preventing influenza (10.6%).

## Editorial Note

Overall influenza vaccination coverage among pregnant women during the 2012–13 influenza season was 50.5%. Vaccination coverage among pregnant women was 47.0%–49.0% for the 2010–11 and 2011–12 influenza seasons (4,5); however, these estimates are not directly comparable because the change in the definition of vaccination status for this most recent season (including changing the measurement of influenza vaccination for pregnant women to July through April and restricting vaccination to receipt before or during pregnancy). Women reporting no health insurance, not working for wages, having fewer than six health-care provider visits since July 2012, or lower socioeconomic status indicators (less education and living below the poverty level) had lower vaccination coverage than other women in the survey. Negative attitudes toward the efficacy or safety of influenza vaccination and having no concern about influenza infection were also associated with lower vaccination coverage. Provider recommendation and offer of influenza vaccination was associated with higher levels of vaccination coverage, even when women reported no health insurance, not working for wages, lower socioeconomic status indicators, a negative attitude toward the efficacy or safety of influenza vaccination, or a lack of concern about influenza infection.

Among women with at least one health-care provider visit, 54.6% reported receiving a provider recommendation and offer of vaccination. In any practice, barriers to providers recommending and offering vaccination might include physician’s concern about time spent discussing the vaccination; administrative and financial issues, such as concern about the up-front cost of ordering vaccines; high costs of storing and maintaining vaccines; not having electronic health records; and organizational challenges of vaccine administration (6–8). Systems supporting provider recommendation and offer, such as standing orders and provider reminder systems, can reduce missed opportunities for vaccination and improve vaccination coverage when implemented with strategies to improve access to vaccination services, such as strategies that reduce patient cost and increase demand (e.g., patient education) (3). Full implementation of the Affordable Care Act might allow access to ACIP-recommended vaccinations, such as influenza vaccination, for pregnant women with no cost sharing when provided by an in-network provider, and thus minimize concerns about vaccination cost. Providers who do not provide vaccinations in their office can recommend vaccination and refer pregnant women to another in-network provider that administers influenza vaccinations.

Pregnant women who were not vaccinated reported concern about the safety risk to their infants and the misconceptions that the vaccination would give them influenza or that vaccination was ineffective as the top reasons for nonvaccination. However, health-care provider recommendation and offer was associated with increased vaccination coverage in all demographic groups. Education messages for pregnant women need to emphasize that vaccination during pregnancy can protect not only pregnant women themselves but also their infants during the first 6 months of life (9). Such messages can be delivered through multiple means, including routine provider education, prenatal consultation, social media, and text messaging (e.g., https://text4baby.org). These efforts might help providers address negative attitudes and misconceptions about vaccination.

What is already known on this topic?

Influenza vaccination coverage among pregnant women increased substantially to approximately 50% during the 2009–10 influenza season, and the increased coverage was sustained during the 2010–11 and 2011–12 influenza seasons.

What is added by this report?

Based on the responses of 1,702 self-selected participants in an Internet panel survey, for the 2012–13 influenza season, 50.5% of pregnant women were vaccinated against influenza, and 72.3% of pregnant women reported receiving a health-care provider recommendation of vaccination. Women who received a provider recommendation and offer of vaccination had higher vaccination coverage than women who received a provider recommendation alone or received no recommendation, even when they had a negative attitude toward vaccination efficacy or the safety of vaccination.

What are the implications for public health practice?

Continued efforts are needed to increase knowledge among pregnant women about the risk for influenza and the safety and efficacy of influenza vaccination for themselves and their infants. Efforts are also needed to increase opportunities for providers to recommend and offer influenza vaccination to pregnant women to protect both them and their infants.

The findings in this report are subject to at least five limitations. First, estimates might be biased if the selection processes for entry into the Internet panel and a woman’s decision to participate in this particular survey were related to receipt of vaccination. Comparing 2010–11 influenza season vaccination estimates from 18 states in both the Internet panel survey and the Pregnancy Risk Assessment Monitoring System (PRAMS), a probability sampling survey, the Internet panel survey estimate for women pregnant at any time during October 2010–January 2011 (50.2%) was similar to the estimate from PRAMS for women who were pregnant in the same period (49.2%) (10). Additional comparisons with PRAMS and other available data sources over multiple seasons are needed to determine whether the more timely Internet panel survey estimates, despite sampling differences, provide valid assessments of trends. Second, the survey was self-administered and not validated by medical record review. Third, the results were weighted to the distribution of pregnant women in the U.S. population, but the study sample did not include women without Internet access. Therefore, it might not be a representative sample of pregnant women, and findings might not be generalizable to all pregnant women in the United States. Fourth, this was a cross-sectional survey. Self-reported vaccination status, attitudes, and provider recommendation and offer were measured at the time of the survey. Interactions that happened before the survey (e.g., choosing a provider with similar attitudes or a change in attitudes because of a provider recommendation or offer) could not be captured by this survey. Finally, the 2012–13 influenza season coverage estimates are not directly comparable with estimates from the 2011–12 and 2010–11 seasons reported previously (4,5) because of the change in measuring vaccination coverage in this season.

Health-care provider recommendation and offer of influenza vaccination were associated with higher vaccination levels among pregnant women. Vaccination programs that include reducing patient cost of vaccination, reducing missed opportunities for vaccination by ensuring vaccination recommendations are provided at each visit, and increasing demand are needed (3). Tailored educational messages should emphasize that vaccination during pregnancy will not only decrease the risk for influenza-related illness and complications in pregnant women themselves, but can also decrease the risk for illness in infants for up to 6 months, while they are too young to be vaccinated (9).

## Figures and Tables

**FIGURE:**
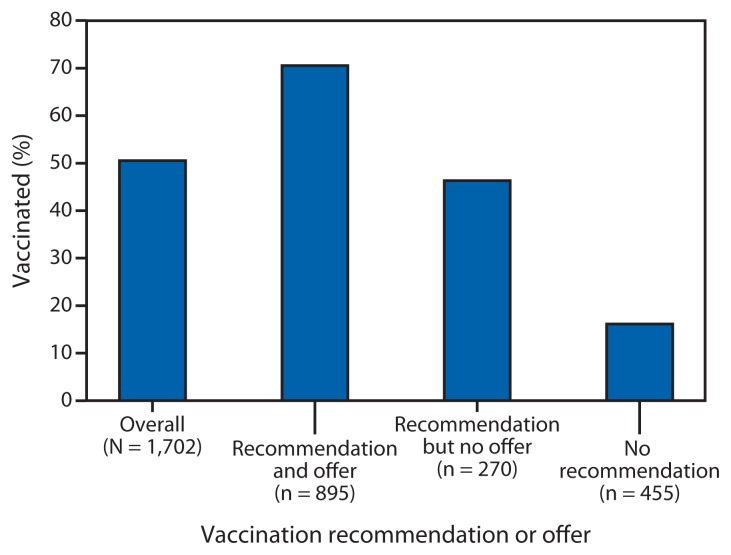
Influenza vaccination before and during pregnancy, overall and by health-care provider recommendation and offer* of influenza vaccination, among women pregnant at any time during October 2012–January 2013 — Internet panel survey, United States, 2012–13 influenza season * Excluded women who did not visit a health-care provider since July 2012 (n = 27) and/or did not respond or did not know whether they received an offer of vaccination (n = 55).

**TABLE 1:** Influenza vaccination coverage among women who were pregnant at any time during October 2012–January 2013, by selected characteristics — Internet panel survey, United States, 2012–13 influenza season

Characteristic	Unweighted no.	Weighted %	Weighted % vaccinated*
**Total**	**1,702**	**100.0**	**50.5**
Vaccinated before pregnancy	239	—	14.6
Vaccinated during pregnancy	638	—	35.9
1st trimester	273	—	15.7
2nd trimester	200	—	10.6
3rd trimester	138	—	8.1
Unvaccinated	776	—	49.5
**Age group (yrs)**			
18–24	477	33.1	48.7
25–34	970	50.5	50.5
35–49	255	16.3	54.1
**Race/Ethnicity**			
White, non-Hispanic	1,093	50.3	52.2
Black, non-Hispanic	175	18.8	45.4
Hispanic	278	23.8	50.1
Other, non-Hispanic	156	7.2	53.1
**Education**			
Less than college degree	844	51.8	43.9
College degree	656	36.8	57.3
More than college degree	202	11.4	58.5
**Married**			
Yes	1,120	62.2	54.8
No	582	37.8	43.5
**Health insurance coverage**			
Any public	659	41.8	50.0
Private/Military only	939	51.7	53.0
No insurance	104	6.5	33.7
**Working status** †			
No	860	50.4	44.7
Yes	842	49.6	56.4
**Poverty status** §			
Below poverty level	404	26.0	41.6
At or above poverty level	1,289	74.0	53.8
**High-risk conditions** ¶			
Yes	613	36.3	57.8
No	1,089	63.7	46.4
**No. of provider visits since July 2012**			
0	27	1.5	—**
1–5	682	41.6	48.0
6–10	598	34.9	53.1
>10	395	21.9	53.1
**Reported provider recommendation and/or offer** ††			
Recommendation and offer	895	54.6	70.5
Recommendation but no offer	270	16.7	46.3
No recommendation	455	28.7	16.1
**Attitude toward efficacy of influenza vaccination** §§			
Negative	430	25.2	9.8
Positive	1,272	74.8	64.2
**Attitude toward safety of influenza vaccination** ¶¶			
Negative	475	28.7	13.0
Positive	1,227	71.3	65.6
**Attitude toward influenza infection** ***			
Not concerned	686	39.5	47.1
Concerned	1,016	60.5	52.8

* Women who reported being vaccinated since July 2012 and being vaccinated either before or during pregnancy were defined as vaccinated. Overall, 2.9% of women reported vaccination after pregnancy and were categorized as unvaccinated during pregnancy. The revised estimates for the 2010–11 and 2011–12 influenza seasons using the 2012–13 definition were 44.0% and 47.6%, respectively (CDC, unpublished data, 2013).

† Those who were employed for wages or self-employed were categorized as working. Those who were out of work, homemakers, students, retired, or unable to work were grouped as not working.

§ Below poverty were defined as a total of annual family income of <$23,283 for a family of four with two minors as of 2012, as determined by the U.S. Census Bureau (information available at http://www.census.gov/hhes/www/poverty/data/threshld).

¶ Conditions associated with increased risk for serious medical complication from influenza, including chronic asthma, a lung condition other than asthma, a heart condition, diabetes, a kidney condition, a liver condition, obesity, or a weakened immune system caused by a chronic illness or by medications taken for a chronic illness.

** Sample size was <30; vaccination coverage estimates were not reliable.

†† Excluded women who did not visit a provider since July 2012 (n = 27) and women who did not respond or did not know whether they received a provider offer (n = 55).

§§ Composite variable created based on responses to two questions regarding attitudes toward influenza vaccination: 1) “Flu vaccine is somewhat/very effective in preventing flu” and 2) “Agree/Strongly agree that if a pregnant woman receives the flu vaccination, it will protect the baby from getting the flu after it is born.” One point was given for each “yes” answer for either of the two questions. Respondents who had a summary score of 1 or 2 were defined as having a “positive” attitude, and those with a summary score of 0 were defined as having a “negative” attitude.

¶¶ Composite variable created based on responses to three questions regarding attitudes toward influenza vaccination: 1) “Flu vaccination is somewhat/ very/completely safe for most adult women,” 2) “Flu vaccination is somewhat/ very/completely safe for pregnant women,” and 3) “Flu vaccination that a pregnant women receives is somewhat/very/completely safe for her baby.” One point was given for each “yes” answer to any of the three questions. Respondents who had a summary score of 2 or 3 were defined as having a “positive” attitude, and those with a summary score of 0 or 1 were defined as having a “negative” attitude.

*** Variable created based on response to a question regarding attitude toward influenza infection: “If a pregnant woman gets the flu, it is somewhat/very likely to harm the baby.” Respondents with a “yes” answer were defined as “concerned,” and respondents with a “no” answer were defined as “not concerned.”

**TABLE 2:** Percentage of pregnant women receiving a health-care provider recommendation for influenza vaccination and influenza vaccination coverage, by provider recommendation and offer and selected characteristics, among women who visited a provider at least once since July 2012 and were pregnant at any time during October 2012–January 2013 — Internet panel survey, United States, 2012–13 influenza season

			Vaccination recommendation or offer					
		
	Reported a provider recommendation		Recommendation and offer		Recommendation but no offer		No recommendation	
				
Characteristic	No.	Weighted %	No.	Weighted %	No.	Weighted %	No.	Weighted %
**Total**	**1,675**	**72.3**	**895** *	**70.5**	**270** *	**46.3**	**455** *	**16.1**
**Age group (yrs)**								
18–24	466	72.2	236	67.5	76	45.3	129	21.0
25–34	956	72.2	519	70.6	154	46.2	261	12.8
35–49	253	72.9	140	75.6	40	49.0	65	16.8
**Race/Ethnicity**								
White, non-Hispanic	1,075	73.3	583	70.8	178	49.5	286	16.6
Black, non-Hispanic	171	69.5	87	66.5	—†	—†	52	20.0
Hispanic	276	71.9	146	72.3	42	39.9	76	12.6
Other, non-Hispanic	153	73.9	79	72.8	—†	—†	41	14.2
**Education**								
Less than college degree	824	69.0	406	65.5	129	41.1	255	13.9
College degree	650	76.9	370	75.9	106	47.2	157	19.9
More than college degree	201	76.9	119	72.2	35	65.2	43	16.9
**Married**								
Yes	1,109	75.4	639	73.5	175	47.0	270	15.7
No	566	67.1	256	64.0	95	45.3	185	16.7
**Health insurance coverage**								
Any public	645	72.1	335	71.7	104	43.3	179	17.4
Private/Military only	930	74.1	522	71.6	151	49.8	236	15.5
No insurance	100	58.9	38	46.4	—†	—†	40	14.2
**Working status** §								
No	840	70.2	420	65.9	142	41.3	245	13.7
Yes	835	74.4	475	74.5	128	51.8	210	18.9
**Poverty status** ¶								
Below poverty level	398	68.7	196	63.1	62	37.7	121	13.2
At or above poverty level	1,268	73.7	696	72.9	206	49.1	330	17.4
**High-risk conditions** **								
Yes	607	78.8	358	73.9	96	54.4	130	19.6
No	1,068	68.5	537	68.1	174	41.5	325	14.8
**No. of provider visits since July 2012**								
1–5	682	67.8	323	69.0	110	49.1	221	16.2
6–10	598	74.2	327	72.6	99	43.8	152	18.1
>10	395	77.7	45	69.7	61	45.1	82	12.3
**Attitude toward efficacy of influenza vaccination** ††								
Negative	422	51.7	147	19.4	57	8.6	206	2.5
Positive	1,253	79.2	748	80.6	213	57.5	249	26.8
**Attitude toward safety of influenza vaccination** §§								
Negative	462	50.6	137	19.4	76	13.7	234	7.7
Positive	1,213	80.9	758	80.1	194	61.2	221	24.8
**Attitude toward influenza infection** ¶¶								
Not concerned	678	70.8	331	68.8	125	44.3	202	15.6
Concerned	997	73.3	564	71.5	145	47.9	253	16.5

* Excluded women who did not respond or did not know whether they received a provider offer of vaccination (n = 55).

† Sample size was <30; vaccination coverage estimates were not reliable.

§ Those who were employed for wages or self-employed were categorized as working. Those who were out of work, homemakers, students, retired, or unable to work were grouped as not working.

¶ Below poverty were defined as a total of annual family income of <$23,283 for a family of four with two minors as of 2012, as determined by the U.S. Census Bureau (information available at http://www.census.gov/hhes/www/poverty/data/threshld).

** Conditions associated with increased risk for serious medical complication from influenza, including chronic asthma, a lung condition other than asthma, a heart condition, diabetes, a kidney condition, a liver condition, obesity, or a weakened immune system caused by a chronic illness or by medications taken for a chronic illness.

†† Composite variable created based on responses to two questions regarding attitudes toward influenza vaccination: 1) “Flu vaccine is somewhat/very effective in preventing flu”; 2) “Agree/Strongly agree that if a pregnant woman receives the flu vaccination, it will protect the baby from getting the flu after it is born.” One point was given for each “yes” answer for either of the two questions. Respondents who had a summary score of 1 or 2 were defined as having a “positive” attitude, and those with a summary score of 0 were defined as having a “negative” attitude.

§§ Composite variable created based on responses to three questions regarding attitudes toward influenza vaccination: 1) “Flu vaccination is somewhat/very/completely safe for most adult women,” and 2) “Flu vaccination is somewhat/very/completely safe for pregnant women,” and 3) “Flu vaccination that a pregnant women receives is somewhat/very/completely safe for her baby.” One point was given for each “yes” answer to any of the three questions. Respondents who had a summary score of 2 or 3 were defined as having a “positive” attitude, and those with a summary score of 0 or 1 were defined as having a “negative” attitude.

¶¶ Variable created based on response to a question regarding attitude toward influenza infection: “If a pregnant woman gets the flu, it is somewhat/very likely to harm the baby.” Respondents with a “yes” answer were defined as “concerned,” and respondents with a “no” answer were defined as “not concerned.”

## Footnotes

* Comparable National Health Interview Survey data for this population will not be available until July 2014.

† Additional information available at http://www.surveysampling.com. The SurveySpot panelists were recruited from Internet sites that host a large number of frequent visitors and diverse Internet traffic. Multiple methods of recruitment were used, including banner ads, direct invitations, pop-ups, and web intercepts. The panel represents approximately 1 million households, and new panelists are continually being recruited; existing panelists are removed from the panel if they have opted-out or have not responded to an invitation within a specified period. A minimal incentive is routinely used to maintain the panel but not for an inducement to participate in a particular survey. Pregnant women panelists in this report were recruited from the SurveySpot panel using two methods: 1) an email invitation from SurveySpot sent to panel members aged 18–49 years, female, and living in the United States; and 2) a pop-up message inviting panel members visiting the SurveySpot website (http://www.surveyspot.com) to answer a series of screening questions and, if eligible, to take the survey.

§ A survey response rate requires specification of the denominator at each stage of sampling. During recruitment of an online opt-in survey sample, such as the Internet panel described in this report, these numbers are not available; therefore, the response rate cannot be calculated. Instead, the survey completion rate is provided.

¶ The sample of pregnant women was weighted to reflect the age group, race/ethnicity, and geographic region of all pregnant women in the United States during 1990–2008. The total population of pregnant women in the United States in 2012 and the distribution of pregnant women by age and race/ethnicity groups was determined based on data for the number of pregnant women in the United States during 1990–2008 (available at http://www.cdc.gov/nchs/data/nvsr/nvsr60/nvsr60_07.pdf). The distribution of U.S. pregnant women age 18–44 years by census region in 2008 was determined based on estimates provided for each state in the Guttmacher Institute’s state data center (available at http://www.guttmacher.org/datacenter).

** Three composite variables were created. First, the influenza vaccination efficacy attitude composite variable was created based on responses to two questions regarding attitudes toward influenza vaccination: 1) “Flu vaccine is somewhat/very effective in preventing flu” and 2) “Agree/Strongly agree that if a pregnant woman receives the flu vaccination, it will protect the baby from getting the flu after it is born.” One point was given for each “yes” answer for either of the two questions. Respondents who had a summary score of 1 or 2 were defined as having a “positive” attitude, and those with a summary score of 0 were defined as having a “negative” attitude. Second, the safety of influenza vaccination attitude composite variable was created based on responses to three questions regarding attitudes toward influenza vaccination: 1) “Flu vaccination is somewhat/very/completely safe for most adult women,” 2) “Flu vaccination is somewhat/very/completely safe for pregnant women,” and 3) Flu vaccination that a pregnant women receives is somewhat/very/completely safe for her baby.” One point was given for each “yes” answer to any of the three questions. Respondents who had a summary score of 2 or 3 were defined as having a “positive” attitude, and those with a summary score of 1 or 0 were defined as having a “negative” attitude. Third, the influenza infection variable was created based on response to a question regarding attitude toward influenza infection: “If a pregnant woman gets the flu, it is somewhat/very likely to harm the baby.” Respondents with a “yes” answer were defined as “concerned,” and respondents with a “no” answer were defined as “not concerned.”
